# Providers’ perspective on vaginal birth after cesarean birth: a qualitative systematic review

**DOI:** 10.1186/s12884-024-06921-1

**Published:** 2024-11-06

**Authors:** Antita Kanjanakaew, Atchareya Jiramanee, Manassawee Srimoragot

**Affiliations:** Department of Obstetric and Gynecological Nursing, Faculty of Nursing, Mahidol University, Bangkok, Thailand

**Keywords:** VBAC, Previous Cesarean, Review, Qualitative Study

## Abstract

**Background:**

Vaginal Birth after Cesarean Birth (VBAC) is a birth mode recommended for reducing repeat cesarean which potentially contributes to adverse outcomes. However, VBAC is not normally practiced in some countries. Providers are an important part of the decision-making process on modes of birth among pregnant individuals. Providers’ perspective on VBAC can influence whether they support or avoid conducting VBAC. This review aimed to explore providers’ perspective on VBAC.

**Methods:**

The comprehensive search was conducted from six databases including PubMed, MEDLINE, Scopus, Cochrane Library, EMBASE, and Google scholar. The studies published in English between 2013 and 2023 were review. The Medical Subject Heading terms for VBAC and perspective were used to search. The eligible studies were selected by the PRISMA flow chart. The initial search yielded 558 articles. After excluding duplicates, articles not retrieved for full-text, and not meeting inclusion and exclusion criteria, eight articles were recruited. Quality appraisal of the studies was performed by the tool of the Joanna Briggs Institute. The meta-aggregation approach was applied to synthesize the findings.

**Results:**

Eight qualitative articles were included in this review, and six themes were developed including (1) different recognition of VBAC, (2) differences of willingness level of conducting Trial of Labor after Cesarean (TOLAC) (the approach attempting to have VBAC), (3) skills and resources needed when performing TOLAC, (4) protocol for recruiting candidacy and TOLAC management, (5) final decision making on VBAC, and (6) onset and duration of providing TOLAC information.

**Conclusion:**

Providers play an important role in influencing individuals’ decision on modes of birth. Providers’ positive recognition and willingness of conducting TOLAC potentially impact successful VBAC rate. However, the lawsuit caused by adverse outcomes from TOLAC/VBAC is a main reason for choosing repeat cesarean.

**Trial registration:**

PROSPERO registration number of this systematic review: CRD42023427662.

**Supplementary Information:**

The online version contains supplementary material available at 10.1186/s12884-024-06921-1.

## Abstract (plain language summary)

Repeat cesarean is a primary contributor to high cesarean rates globally. Vaginal Birth after Cesarean Birth (VBAC) is a vaginal birth of individuals with previous cesarean. While some countries have high successful VBAC rates, many countries have very low VBAC rates and do not support a vaginal birth in subsequent pregnancy among individuals with previous cesarean. Healthcare providers especially obstetricians and nurse-midwives play an important role in individuals’ decision on mode of birth, so do individuals with previous cesarean. Healthcare providers’ perspective on modes of birth among individuals with previous cesarean is very essential to support or not support individuals to have VBAC. In high VBAC rate countries, healthcare providers think VBAC is an optimal option for individuals with previous cesarean and have high confidence to do VBAC. On the other hand, healthcare providers in low VBAC rate countries think VBAC can cause a catastrophic birth outcome as uterine dehiscence and they are scared of being sued from the adverse outcome. One approach to support VBAC, most healthcare providers suggest that individuals with previous cesarean should be educated about VBAC right after the first cesarean or first antenatal visit because VBAC is not a common mode of birth for individuals in some countries and they need time to make a decision on mode of birth for their subsequent pregnancy.

**Supplementary Information:**

The online version contains supplementary material available at 10.1186/s12884-024-06921-1.

## Introduction

Cesarean rates have been rising worldwide. The World Health Organization recommended the optimal cesarean rate should be between 15 and 20% and there is no significant decrease in maternal and neonatal mortality and morbidity if the cesarean rate is over than the recommended number [1, 2]. On the other hand, having cesarean can elevate risk for adverse maternal and neonatal health outcomes when cesarean is medically unnecessary used [3, 4]. The global survey examining cesarean rates of 154 countries worldwide from 2010 to 2018 showed that global cesarean rate was 21.1%, and this rate is expected to reach almost 30% by 2030 [5]. This high global cesarean rate could indicate that there might be unnecessary medically cesarean in settings.

When investigating the main contributor to higher cesarean rate, the previous studies showed that the repeat cesarean as well as history of myomectomy is the largest contributor to high cesarean rate in a setting [6–10]. Vogel and colleagues assessed the cesarean trend in 21 countries across the world by using Robson classification system found that almost all countries had higher cesarean rate and previous cesarean was a significant determinant of overall cesarean rates [11]. Kanjanakaew et al. also conducted the literature review focusing on cesarean in Asia and found that repeat cesarean is a primary contributor to higher cesarean rate in Asian countries [6].

Although repeat or elective cesarean among individuals with previous cesarean is more in controlled compared to birth vaginally [12], it potentially leads to adverse pregnancy and birth outcomes to both individuals and their children [13]. The prior studies investigating the outcomes of individuals having repeat cesarean showed these individuals were more likely to have severe adhesions, blood transfusions, and bladder injured [13–15]. Preterm birth in the subsequent pregnancy is also more common in individuals having repeat cesarean [15]. The long-term birth outcomes of children born by having repeat cesarean in previous study were asthma and learning disability [16]. Moreover, the population-based study in Ohio examining breastfeeding initiation among individuals with previous cesarean categorized into three groups (a) repeat cesarean, b) successful vaginal birth after cesarean (VBAC), and c) unsuccessful VBAC) revealed that individuals with successful VBAC and unsuccessful VBAC had a greater breastfeeding initiation rate compared with individuals with repeat cesarean [17]. Furthermore, when comparing cost-effectiveness between repeat cesarean and VBAC in Ireland, VBAC was significantly less expensive than repeat cesarean [18].

Individuals with previous cesarean have two options for giving birth in the subsequent pregnancy, including (a) repeat cesarean, or (b) vaginal birth known as VBAC. The attempt to have VBAC is called Trial of Labor after Cesarean (TOLAC) [19]. VBAC is an individual’s vaginal birth following cesarean in a prior pregnancy [20], and it can effectively reduce high cesarean rates [21]. Some countries in Europe showed they had high successful VBAC rates which can lessen cesarean rates in various settings [22–24]. Although VBAC is common in several countries, conducting TOLAC to have VBAC is not normally practiced in some countries [23, 25, 26]. In Thailand, individuals with previous cesarean were scheduled to have repeat cesarean, and VBAC was not common and recommended in their setting [27]. Another study in Bhutan showed repeat cesarean was the only choice for individuals with previous cesarean because no protocol for TOLAC was utilized in their setting, and this leads to high cesarean rate among individuals with previous cesarean [28]. Additionally, a qualitative study investigating clinician’s view on VBAC in low VBAC rate countries showed that most providers in those countries thought conducting TOLAC is a procedure required clinical expertise or skills [23, 25]. Other reasons for not performing TOLAC are need of collaboration across professionals for conducting TOLAC and requiring a significant amount of time for counselling and providing the information about TOLAC/VBAC to individuals as well as their partner or family members [22, 23, 29, 30].

Providers’ perspective on VBAC is a vital contribution which influences care for pregnant individuals as well as making a decision on mode of birth. The previous literature review showed that individuals’ preference of birth mode was mainly influenced by medical staff’s recommendation or physicians’ opinion [12]. This can imply that healthcare providers are a significant person influencing individuals’ mode of birth. Discrepancy of perspective on VBAC may impact differences of VBAC rate across countries. While several studies have explored perspectives on VBAC among providers working on maternal and neonatal care, the comprehensive synthesis of these findings is lacking. In addition, the previous literature review exploring perspective on VBAC mainly focus on pregnant individuals’ perspective. This review aimed to explore providers’ perspective on VBAC, in which understanding providers’ perspective on VBAC will allow the development of the optimal intervention to cultivate positive perspective on TOLAC and to support VBAC.

## Methods

This systematic review recruited qualitative studies meeting the inclusion criteria between January 2013 and July 2023. This timeframe was selected to understand the contemporary perspective on VBAC which has been encouraged to reduce cesarean rate.

The six databases used to search were PubMed, MEDLINE, Scopus, Cochrane Library, EMBASE, and Google scholar. The Medical Subject Heading (MeSH) terms for VBAC include VBAC OR “Vaginal birth after cesarean” OR “Vaginal birth after caesarean” OR “TOLAC” OR “Trial of labor after cesarean” OR “Trial of labour after caesarean”, and MesH tems for perspective include perspective OR view OR viewpoint OR aspect. In term of providers, the MesH terms include “health care provider” OR provider OR midwife OR midwives OR obstetrician OR doctor OR physician OR MD.

The inclusion criteria were: (a) perspectives on VBAC or birth mode were report, (b) all healthcare providers working in the maternity unit were a sample of a study, (c) a qualitative study, (d) written in English, and (e) published in a peer-review journal. Exclusion criteria were (a) sample not mentioned (neither providers nor pregnant individuals) and (b) a study presented in conference, expert’s opinion. In addition, this study was registered in the PROSPERO to confirm that there have not been any existing systematic reviews on this topic. The PROSPERO registration number is CRD42023427662.

**Fig. 1:**
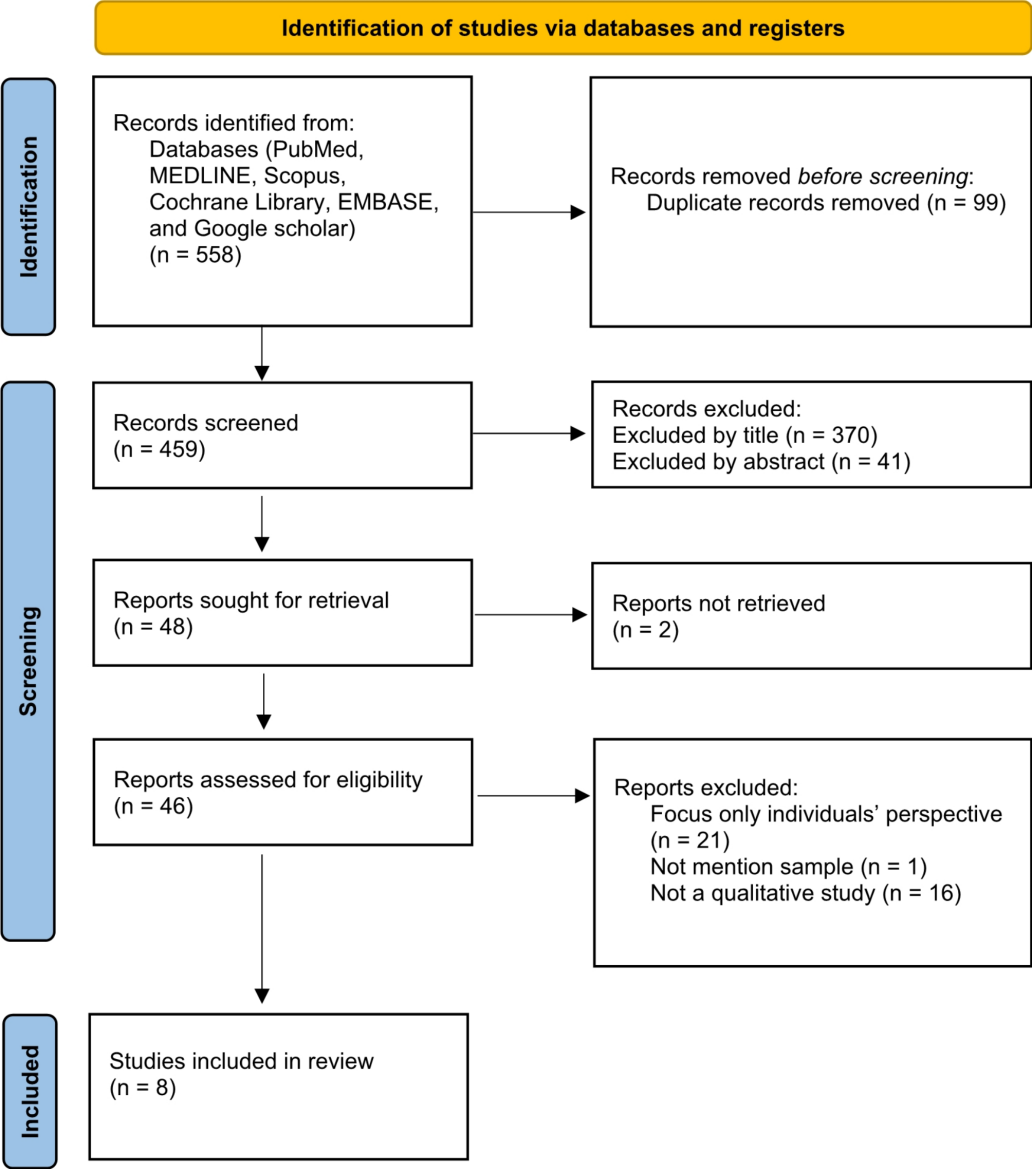
PRISMA diagram

The initial search yielded 558 articles. Once duplicates were excluded and the titles as well as abstract of the remaining articles were screened, resulting in 48 articles for full-text screening. After excluding articles not retrieved for full-text and not meeting inclusion and exclusion criteria, the total of eligible for this review was eight articles (see PRISMA flow chart in Fig. 1).

Quality appraisal was performed using The Joanna Briggs Institute (JBI) Critical Appraisal tools for use in JBI Systematic Reviews by two authors (see Table 1) [31]. Any discrepancy was resolved by the third author’s appraisal. The detailed quality appraisal of each study is available upon request. All included studies had JBI score higher than 80% (high quality) and were reviewed. Data extraction included author, year of publication, country(ies), study design, and types of providers, and findings (providers’ perspective on VBAC). The level of plausibility of each eligible study was also assessed during data extraction process.

**Table 1:** Appraisal of Included Studies^a^

Citation	Q1	Q2	Q3	Q4	Q5	Q6	Q7	Q8	Q9	Q10
Chan, Bronson, & Cantor (2015) [32]	Y	Y	Y	Y	Y	U	U	Y	Y	Y
Firoozi, Tara, Mazloum, &Latifnejad (2021) [33]	Y	Y	Y	Y	Y	U	U	Y	Y	Y
Foureur et al. (2017) [34]	Y	Y	Y	Y	Y	Y	U	Y	Y	Y
Jimenez-Zambrano et al. (2022) [29]	Y	Y	Y	Y	Y	U	U	Y	Y	Y
Lundgren et al. (2016) [25]	Y	Y	Y	Y	Y	Y	Y	Y	Y	Y
Lundgren et al. (2015) [22]	Y	Y	Y	Y	Y	Y	Y	Y	Y	Y
Lundgren, Morano, Nilsson, Sinclair, & Begley (2020) [23]	Y	Y	Y	Y	Y	Y	Y	Y	Y	Y
Munro et al. (2017) [35]	Y	Y	Y	Y	Y	Y	U	Y	Y	Y

^a^Q1 = Congruity between the stated philosophical perspective and the research methodology

Q2 = Congruity between the research methodology and the research question or objectives

Q3 = Congruity between the research methodology and the methods used to collect the data

Q4 = Congruity between the research methodology and the representation and analysis of the data

Q5 = Congruity between the research methodology and the interpretation of the results

Q6 = Statement locating the researcher culturally or theoretically

Q7 = Statement of the influence of the researcher on the research

Q8 = Representation of the participants and their voices

Q9 = Ethical approval by an appropriate body

Q10 = Relationship between the conclusions and analysis or interpretation of the data

Y; Yes, N; No, U; Unclear

Data Synthesis was done by applying meta-aggregation. This process have been utilized worldwide [31], which would begin with extraction of all findings with illustration of each finding from the eligible studies. Then, at least two findings can develop a category for findings. Lastly, synthesized findings were developed from the categories (at least two categories can develop one synthesized finding). All data analyzed during this study are included in the supplementary file.

## Results

Eight qualitative articles were included in this systematic review. The quality appraisal of included studies is presented in Table 1. Of eight, half of them were conducted by individual interview. The sample in this review were health care providers including obstetricians [22, 23, 25, 29, 32–35], midwives [22, 23, 25, 32–35], general physicians [23, 25, 35], family physicians [35], neonatologists [25], nurses [35], anesthetists [35], and health service decision maker [35]. All recruited studies interviewed obstetricians, and seven studies interviewed both obstetricians and midwives [22, 23, 25, 32–35]. Other details are presented in Table 2. Six themes were identified after synthesizing the data regarding providers’ perspective on VBAC (see Table 3).

**Table 2:** The articles included in the review and themes identified

Authors (year)	Country(ies)	Study Design	Sample and Sample Size	Theme
Chan, Bronson, & Cantor (2015) [32]	United States	Individual interview	5 individuals and 6 providers (5 obstetricians and 1 Midwife)	1,2,6
Firoozi, Tara, Mazloum, &Latifnejad (2021) [33]	Iran	Individual interview	13 individuals and 12 providers (5 obstetricians and 7 midwives)	2,6
Foureur et al. (2017) [34]	Australia	Focus group	3 obstetricians and 15 midwives	1,2,3,4,6
Jimenez-Zambrano et al. (2022) [29]	Guatemala	Individual interview	10 obstetricians and 20 individuals	1,3,4,5,6
Lundgren et al. (2016) [25]	Ireland, Italy and Germany	Focus group	71 clinicians (35 midwives, 29 obstetricians, 6 doctors, and 1 neonatologist)	1,2,3,5,6
Lundgren et al.(2015) [22]	Finland, Sweden, and Netherland	Individual interview and focus group	44 clinicians (26 midwives and 18 obstetricians)	1,2,3,4,5,6
Lundgren, Morano, Nilsson, Sinclair, & Begley (2020) [23]	Germany, Italy and Ireland (low VBAC countries) and Sweden, Netherlands and Finland (high VBAC countries)	Individual interview and focus group	115 clinicians (62 midwives and 53 obstetricians/physicians) and 73 individuals	1,2,3,4,5,6
Munro et al. (2017) [35]	Canada	Individual interview	35 Providers (3 family physicians, 4 midwives, 4 obstetricians, 3 general practitioner, 7nurses, 1 anesthetist, and 13 health service decision makers)	1,3,4,5,6

**Table 3:** Themes identified during the review

No.	Themes
1.	Different recognition of VBAC among providers
2.	Differences of willingness level of conducting TOLAC
3.	Skills and resources needed when planning to do TOLAC
4.	Protocol for recruiting candidacy and TOLAC management
5.	Final decision making on VBAC
6.	Onset and duration of providing information about TOLAC

### Theme 1 Different recognition of VBAC among providers

Several providers acknowledge VBAC as an appropriate mode of birth for individuals with previous cesarean especially who are healthy.

*“For the patient’s well-being.having a normal delivery is of lower risk.”* (Physician in Guatemala) [29].

Additionally, the providers report VBAC can elevate individuals’ confidence. Giving birth vaginally makes individuals proud of themselves.

*“They really feel like*,* ‘You see*,* I can do this!’ and they are very pleased with it. That is also an important reason”* (Obstetrician in Netherland) [22].

When considering the cost of mode of birth, VBAC is less expensive than repeat cesarean, and individuals will be satisfied with the cost.

*“I think it will favor the cost of the patient*,*”* and *“yes*,* I mean*,* a vaginal delivery [VBAC] is theoretically cheaper than a cesarean delivery.”* (Physician in Guatemala) [29].

However, some providers thought TOLAC potentially contributes to catastrophic outcome as uterine rupture. And, this adverse outcome is related to maternal and neonatal mortality or morbidity.

“*Aside from failing*,* having to have a repeat C-section and uterine rupture are really the only complications. It’s just that uterine rupture has going along with it a laundry list of significant complications as a result*.” (Obstetrician in the United States) [32].

### Theme 2 Differences of willingness level of conducting TOLAC

A provider in Iran stated few providers are willing to do the TOLAC although individuals are sometimes interested in having VBAC for mode of birth of their subsequent pregnancy.

*“I tell them that you can have a vaginal delivery. They say that our gynecologist does not accept it*,* which means that there are only a few gynecologists who are willing to assume the responsibility of doing VBAC.”* (Obstetrician in Iran) [33].

Another main finding which contributes to avoid performing TOLAC among providers especially obstetricians is fear of lawsuit. Maternity providers are normally sued when adverse birth outcomes occur either among mothers or neonates. To protect themselves from lawsuit, providers tend to avoid performing TOLAC which might unexpectantly have catastrophic outcomes.

*“The medico-legal issues in Ireland are probably adverse compared with Sweden*,* where there is absolutely no chance of you being sued over a VBAC. A high VBAC rate with a poor neonatal outcome is not acceptable.”* (Obstetrician in Ireland) [25].

However, there is a study revealed nurse-midwives have a program which support VBAC among individuals with previous cesarean. The pregnant individuals are provided the TOLAC/VBAC information as an alternative birth mode.

*“And I’ve noticed that even in the last couple of years since we’ve had that ‘‘Towards Normal Birth’’ midwife*,* what I would see happening is that women would want a VBAC*,* they would come through the clinic*,* get to their thirty-sixth week visit with the doctor and. Change their mind. Now*,* I find that with our ‘‘Towards Normal Birth’’ midwife*,* she’s also there and she’s informing the women [about their VBAC options]”* (Midwife in Australia) [34].

### Theme 3 Skills and resources needed when planning to do TOLAC

Although conducting TOLAC is relatively similar to natural childbirth, it is needed providers with expertise in TOLAC. It is because they can handle with what will happen during the TOLAC.

*“Nobody can tell what will happen during a trial of labour (TOL)*,* so we should say that a TOL is possible*,* but only if we have staff who are not overworked and exhausted.”* (Obstetrician in Italy) [25].

Besides expertise, providers’ confidence is also needed for the greater use of TOLAC. This confidence usually comes from their past experiences of conducting TOLAC.

*“I feel confident looking after a VBAC lady . Although my main concern at the back of my mind is the monitoring .‘because she’ll need that extra monitoring . so in the back of my mind I always try and think that active labour and being upright and [leaning] forward for a VBAC is more . Is really important.”* (Midwife in Australia) [34].

Not only professional skill but also interprofessional collaboration are required to taking care of individuals having TOLAC. Communication and collaboration across interprofessional are very crucial and can raise quality of maternity care.

*“Good collaboration [is needed] between clinical midwives*,* physician assistants and gynaecologists when speaking of interpretation of the CTG [cardiotocograph]. So if you are not in the room or you cannot see the CTG*,* you will be summoned [if CTG patterns deviate].”* (Obstetrician in New Zealand) [22].

Hospital resources for giving birth is also important when individuals with previous cesarean having TOLAC. These resources are not only resources for support giving birth by VBAC but also managing the complications during VBAC or unsuccessful VBAC which needs to have repeat cesarean.

*“It is deficient*,* we don’t have many stretchers*,* there is no surgical area for any complications*,* we do not have enough space”* (Obstetrician in Guatemala) [29].

### Theme 4 Protocol for recruiting candidacy and TOLAC management

VBAC is not a perfect mode of birth for all individuals with prior cesarean [22]. Some individuals might have more risks for complication rather than getting better outcomes from VBAC. Therefore, protocol for choosing good TOLAC candidates should be clearly announced.

*“We usually determine the approximate [fetal] weight through ultrasound.we also assess her pelvis.and we are constantly monitoring her during labor and delivery to see how she is doing. If at some point we notice that it is not working*,* we immediately stop it and we don’t give her any more time.”* (Obstetric in Guatemala) [29].

During the labor progressing, individuals who is experiencing TOLAC should be closely monitor to detect adverse birth outcomes of individuals and their children. However, the study from high VBAC rate countries showed individuals with previous cesarean should closely monitor, but do not let the complications be the main awareness.

*“Continuous CTG according to protocol is recommended. However*,* the difficulty with that is the risk for uterine rupture is 1:1000 and so very low. Then I wonder if we should really tie every woman to the bed with fetal monitoring attached and not even allow her to shower for half an hour. I am a little flexible in this.”* (Obstetrician in New Zealand) [22].

TOLAC is also a long process. Individuals having TOLAC should be motivated to have confidence to get through the labor process.

*“It is important that women [during labour] are supported in their goal– ‘this time*,* I am going to do it myself’ – and that professionals help them with that.”* (Midwife in New Zealand) [22].

### Theme 5 Final decision making on VBAC

Beside family members potentially influencing individuals’ decision making on VBAC, social media as Facebook can also impact individuals’ final decision on VBAC.

*“They have seen all this [modes of birth] from Facebook*,* from social networks and they publish everything.”* (Doctor in Guatemala) [29].

There was the study mentioning that individuals should participate in the decision-making process, however, healthcare professionals should make a final decision because they are expertise and have medical knowledge.

*“That’s about the same thing as if I decide how the plumber should place the pipes in my home.”* (Midwife in Sweden) [22].

### Theme 6 Onset and duration of providing information about TOLAC

Individuals in some countries especially where VBAC is not common have not heard about TOLAC or VBAC. They usually go through repeated cesarean. Therefore, before the decision-making process, the accurate TOLAC information should be provided.

*“We have to educate mothers*,* prepare pamphlets*,* and hold training classes. We can give enough audio-visual information even through radio and television. Because when the information is given through the public media or even newspapers*,* women and even their spouses and parents are informed about this process*,* and it will be thus very influential in mother’s decision .”* (Obstetrician in Iran) [33].

Additionally, the TOLAC information should be provided as soon as possible or even right after primary cesarean. This is because counselling about TOLAC/VBAC which is needed TOLAC information is time-consuming [32].

*“I think you need to start educating people now if they’ve just had their primary section for whatever reason. By the time you hit that second pregnancy*,* they often have made up their [mind]”* (Urban Family Physician in Canada) [35].

## Discussion

Findings of this systematic review and synthesis found that there are differences of recognition of VBAC and willingness of conducting TOLAC among providers. Also, the sample of the articles showed their thoughts about factors influencing successful VBAC. The findings of this review are discussed by themes.

### Different recognition of VBAC among providers

The eligible studies found that midwives typically consider VBAC as an optimal option or the primary alternative for individuals with previous cesarean [25, 29, 35], while some obstetricians thought it can contribute adverse outcomes as uterine dehiscence [32, 34]. This different recognition of VBAC might be because each healthcare profession is differently informed or trained in TOLAC/ VBAC. Keedle et al. mentioned that most midwives typically promote physiological birth and advocate individuals with previous cesarean for having TOLAC [36]. They, therefore, recognize TOLAC as an optimal choice for individuals with history of cesarean to have VBAC [36]. Additionally, the prior study showed that greater proportion of midwife-attended births was associated with using TOLAC and higher successful VBAC rate [37]. Also, White et al. found that midwife-led model of the antenatal care is significantly associated with higher VBAC rate [38]. However, there was a study revealed that there was no difference in successful VBAC rate between types of providers (e.g., obstetricians, midwives) [39]. The indifferent rates of successful VBAC might be because this study focused only low-risk pregnant individuals who were more likely to have successful VBAC [39].

Moreover, the studies from high VBAC rate countries (e.g., Finland, Netherland, Sweden) usually considered VBAC as the first alternative for those with previous cesarean. Yet, the low VBAC rate countries (e.g., Germany, Italy, and Ireland) did not always consider VBAC as the first choice [23]. Care culture is important for birth planning of individuals, meaning that some care cultures always support vaginal birth while some do not [23]. Therefore, to improve VBAC rate in a country, care culture in that country should be shaped to target toward vaginal birth.

### Differences of willingness level of conducting TOLAC (#6)

In term of reluctant to perform TOLAC, we found that midwives in the eligible studies usually mentioned that not all obstetricians support VBAC or were willing to do TOLAC [33, 34]. One main reason for avoiding conducting TOLAC among obstetricians is fear of lawsuit from which is resulted adverse birth outcomes (e.g., maternal and neonatal mortality or maternal and neonatal morbidity). Fear of litigation is an essential factor for obstetricians to decide performing surgical birth or cesarean [40]. Deng et al. mentioned that obstetricians protect them from the lawsuits which are caused by complication during the vaginal birth by choosing cesarean [41]. In contrast, midwives are seen to be willing to perform TOLAC [34]. This willingness of midwives might be because midwives are trained to be skilled in TOLAC/VBAC and manage the risks of TOLAC. Although they are not authorized to perform cesarean when individuals are not able to give birth vaginally, midwives can assess the need for cesarean and transfer individuals to get the optimal advanced care [42]. In addition, midwives working in diverse settings including home births or birth centers, might perceive less institutional pressure or fear of litigation, influencing their willingness towards TOLAC. In contrast, obstetricians usually work within hospital systems might face stricter policies or concerns about litigation in case of adverse outcomes during VBAC. The findings of recruited studies can provide a glimpse about differences of willingness between provider types which obstetricians have some concerns about lawsuits while midwives do not. One possible reason might be individuals under midwifery care are typically healthier and less complication which tend to have less chance of having severe birth outcomes.

### Skills and resources needed when planning to do TOLAC (#6)

Of eight, three studies mentioned that TOLAC required expertise and confidence [22, 25, 34], meaning that providers performing TOLAC need to be trained and skilled. When being trained and having experiences of conducting TOLAC, they are normally confident to do TOLAC. Although VBAC can be considered as a natural birth, it still needs a support from providers who are trained and skilled to assess and monitor individuals’ labor progress as well as prevent adverse outcomes.

Beside personal skills and confidence needed, collaboration and communication in a team are also essential to improve VBAC rate. Studies showed that good collaboration and effective communication between interprofessional are a part of successful VBAC rate especially in high VBAC rate countries [22, 23]. The prior US studies compared using of cesarean between settings with and without midwife-physician interprofessional model existing by using Robson Ten-Group classification system. This study also found that cesarean rates in the setting with midwives (interprofessional setting) were lower than those without midwives, while the VBAC rates in the setting with midwives were higher than those without midwives [43]. To plan for performing TOLAC, all involved providers should prepare together. Not only obstetricians and midwives but also, aestheticians and other providers should communicate and collaborate within a team, especially when birthing of an individual needs the advanced care due to failed VBAC.

In addition, two eligible studies found that the providers believe TOLAC should be done when the operating room scheduled to prepare for helping individuals when their uterus ruptures [29, 35]. Tertiary hospitals or hospitals with sufficient resources might be optimal to be a setting supporting VBAC. However, in a rural hospital or a setting with limited resources, the providers said they needed to make it work through the TOLAC process by having a good communication ahead and collaboration with providers team [35]. This can indicate that good communication with team and planning ahead is more important than resources used for performing TOLAC.

### Protocol for recruiting candidacy and TOLAC management (#5)

Two eligible studies revealed TOLAC protocols or guidelines are required for assessment TOLAC candidacy and TOLAC management [22, 29]. Although many studies investigating tools for predicting successful VBAC [44, 45], few countries have a protocol or guideline for determining individuals being able to have TOLAC and TOLAC management [46]. Clinical care guideline is essential because it is a framework for clinically making a decision and directing the best practice which are developed from literature review, research outcome, or clinical standards of care [47, 48]. Having protocols or guidelines, therefore, enables providers to properly choose an optimal care for an individual especially in a country where TOLAC is not commonly used. Besides enhancing quality of maternity care, the obstetrics guidelines can prevent provider from obstetrics malpractice which results in lawsuit [49].

In term of TOLAC management, providing care for individuals with previous cesarean during labor is similar as the management of normal birth among individuals without previous cesarean, however, it needs some extra monitoring and motivation for giving birth vaginally [22, 29, 34, 35]. This extra monitoring is able to detect and prevent adverse event as uterine dehiscence which leads to maternal/neonatal mortality and/or morbidity. Additionally, motivation is needed during labor because this stage is a typically long and challenging process [22].This result is consistent with the qualitative study exploring individuals’ experience of VBAC in Cyprus. The study showed that individuals with previous cesarean who attempted vaginal birth had positive experience during labor when they received support and were encouraged by midwives (e.g., breathing exercise, walking, or using birth ball) during the intrapartum period [50].

### Final decision making on VBAC (#5)

Although making a decision process for VBAC should be the involvement of both providers and individuals, the final decision on mode of birth made by either of them can be a different. The eligible studies showed providers are the one who can make a decision in high VBAC rate countries while individuals or their significant ones mostly made a decision on birth plan after previous cesarean in low VBAC rates countries [22, 23, 25, 35]. This can reflect that providers play a crucial role on making a final decision on VBAC. If they support pregnant individuals to have VBAC, the VBAC rate in that country will be high. On the other hand, if providers do not recommend individuals to have VBAC, the VBAC rate in that country tends to be low. This is consistent with the prior study conducting in Thailand where the VBAC rate is not high because their institution do not recommend conducting TOLAC [27].

Moreover, the finding can point that whoever making a final decision (providers vs. individuals and their significant ones) potentially influences birth plan and VBAC rate in a setting. Individuals and their family in low VBAC countries might not be familiar with VBAC, so they are less likely to think about VBAC when considering about birth mode. This indicates that TOLAC/VBAC information should be introduced to not only pregnant individuals but also family. Additionally, when VBAC is not common in countries, people in that country do not know and do not support to get VBAC. The previous study exploring final decision making and birth mode of individuals with previous cesarean in Peru, where VBAC rate is not high, found that over one third did not get any option (neither VBAC nor repeat cesarean). Finally, only 17.65% of these individuals had VBAC [51]. This highlights how presenting multiple birth options or restricting choices can influence individuals’ final decision making on birth mode and the likelihood of VBAC.

### Onset and duration of providing information about TOLAC (#8)

All studies mentioned that counselling for VBAC should be done as soon as possible either right after previous cesarean or first antenatal visit of individuals with previous cesarean. This is because TOLAC is quite difficult topic especially for novices which needs time to be provided the unbiased information. Besides, language used for explaining about TOLAC/VBAC is complicated for people who are not common with the medical terms [29]. Also, counselling is a time-consuming process which individuals need to cooperate all information provided by providers to have an optimal final decision. Counselors, therefore, should dedicate time to discuss about TOLAC/VBAC plan because individuals usually require more time to consider whether they should go through TOLAC process or not [30]. A practical evidence-based approach for VBAC also illustrated that women with previous cesarean should be counselled about risks and benefits of VBAC and repeat cesarean since the early antenatal visit. The aim of the counselling is to provide sufficient information and pregnant individuals will fully understand their delivery options [52].

However, the prior study recommended antenatal counselling about performing repeat cesarean for individuals with multiple previous cesareans to avoid adverse outcomes from conducting TOLAC [53]. This might indicate that individuals with multiple previous cesareans especially in low resource settings were more likely to have high risk for adverse birth outcome, so providers would provide antenatal counselling to prevent catastrophic outcome as uterine rupture.

This systematic review of the qualitative studies of providers’ perspective on VBAC has the strength which is having perspectives of providers in both high and low VBAC rates countries. This can provide the glimpse of how providers’ perspective influences VBAC rate on a global scale. Also, this is an initial study exploring the providers’ perspectives regarding VBAC, while previous studies have predominantly focused on individuals’ perspectives.

However, there are several potential limitations of this review. First potential limitation is all studies did not clearly state if they explored perspective on VBAC of individuals with multiple previous cesareans or VBAC of individuals with one or two previous cesareans. Greater number of previous cesareans are more likely to have adverse birth outcomes which are typically concerned. Moreover, most studies in this review did not mention level of hospitals or clinical settings at where providers work. There might be heterogeneity which can affect providers’ perspective on VBAC and readiness for conducting TOLAC. One of potential limitations is a study exploring perspective on VBAC among providers from East and South-East Asian countries is lacking. This may limit generalizability of these findings to perspective on VBAC among providers in East and South-East Asia. Therefore, the perspective on VBAC among providers in East and South-East Asian countries should be explored in order to know if there are differences of perspective on VBAC or maternity care culture among providers in East and South-East Asian countries and those in other countries. Also, the sample size of particular studies in this review were small, and this might lead to insufficient representation of codes and themes. Lastly, limiting the review to studies published in English may introduce both language and publication bias, limiting the generalizability of our results, and potentially overlooking important research available in other languages.

### Implications

To cultivate positive perspective on VBAC, this birth mode should not be seen as a contributor to adverse birth outcomes. Also, consent form for TOLAC is needed prior the procedure to protect providers from the lawsuit caused by unexpectedly adverse birth outcomes. In addition, to reduce cesarean rate and raise successful VBAC rate, providers should be trained for conducting TOLAC which will increase their confidence in performing VBAC. Having standardized TOLAC protocols enables providers to offer optimal care, especially in countries where TOLAC is not widely practiced. Besides, having standardized guidelines for determining TOLAC candidacy allows providers to screen pregnant individuals having low risk for adverse birth outcome prior conducting TOLAC. Additionally, planning for TOLAC should be considered immediately after the first cesarean or first antenatal visit in order to counsel and provide information about TOLAC to pregnant individuals and their significant ones. The future research should compare successful VBAC rate between individuals counselled about VBAC promptly after the first cesarean and those counselled at the first antenatal visit.

## Conclusion

Cesarean rate is globally increasing especially in a group of individuals with previous cesarean. VBAC is an alternative birth mode for these individuals to have vaginal birth, which may reduce cesarean rate. Healthcare providers play an important role in influencing individuals’ decision on mode of birth. Differences of recognition on VBAC and willingness of conducting TOLAC among providers potentially have an effect on successful VBAC rate in different healthcare settings. The lawsuit caused by adverse birth outcomes from conducting TOLAC is a main reason of choosing elective cesarean instead of TOLAC. To support VBAC, it is essential to train healthcare providers and ensure well-coordinated collaboration among the healthcare team. Lastly, TOLAC counselling should be performed early either after the first cesarean or first antenatal visit.

## Electronic Supplementary Material

Below is the link to the electronic supplementary material.

Supplementary Material 1

## Data Availability

All data analyzed during this study are included in the manuscript (see the supplementary file).

## Abbreviations

VBAC: Vaginal birth after cesarean birth
TOLAC: Trial of labor after cesarean

## References

[1] Betran AP, Torloni MR, Zhang J, Ye J, Mikolajczyk R, Deneux-Tharaux C, et al. What is the optimal rate of caesarean section at population level? A systematic review of ecologic studies. Reprod Health. 2015;12(57):1–10. 26093498 10.1186/s12978-015-0043-6 PMC4496821

[2] Ye J, Betrán AP, Vela MG, Souza JP, Zhang J. Searching for the Optimal Rate of Medically Necessary Cesarean Delivery. Birth. 2014;41(3):237–44. 24720614 10.1111/birt.12104

[3] Antoine C, Young BK. Cesarean section one hundred years 1920–2020: the Good, the Bad and the Ugly. J Perinat Med. 2020;49(1):5–16. 32887190 10.1515/jpm-2020-0305

[4] Chongsuvivatwong V, Bachtiar H, Chowdhury ME, Fernando S, Suwanrath C, Kor-anantakul O, et al. Maternal and fetal mortality and complications associated with cesarean section deliveries in teaching hospitals in Asia. J Obstet Gynaecol Res. 2010;36(1):45–51. 20178526 10.1111/j.1447-0756.2009.01100.x

[5] Betran AP, Ye J, Moller AB, Souza JP, Zhang J. Trends and projections of caesarean section rates: global and regional estimates. BMJ Glob Health. 2021;6(6):1–8. 10.1136/bmjgh-2021-005671 PMC8208001 34130991

[6] Kanjanakaew A, Driessnack M, Tilden EL. Cesarean Birth among Women Birthing in Asia: A Literature Synthesis using the Robson 10-Group Classification System. Asian J Pregnancy Childbirth. 2022;15–31.

[7] Kankoon N, Lumbiganon P, Kietpeerakool C, Sangkomkamhang U, Betrán AP, Robson M. Cesarean rates and severe maternal and neonatal outcomes according to the Robson 10-Group Classification System in Khon Kaen Province, Thailand. Int J Gynecol Obstet. 2018;140(2):191–7. 10.1002/ijgo.12372 29094345

[8] Montoya A, Georgiou C. Caesarean section analysis using the Robson classification in two major hospitals in Victoria: an observational study [Internet]. Preprints; 2020 Aug [cited 2021 Jun 16]. https://www.authorea.com/users/353298/articles/477255-caesarean-section-analysis-using-the-robson-classification-in-two-major-hospitals-in-victoria-an-observational-study?commit=8369daa64b4dae120f023ba9e326c4649e99b53f

[9] Roberge S, Dubé E, Blouin S, Chaillet N. Reporting Caesarean Delivery in Quebec Using the Robson Classification System. J Obstet Gynaecol Can. 2017;39(3):152–6. 28343556 10.1016/j.jogc.2016.10.010

[10] La Verde M, Cobellis L, Torella M, Morlando M, Riemma G, Schiattarella A, et al. Is Uterine Myomectomy a Real Contraindication to Vaginal Delivery? Results from a Prospective Study. J Invest Surg. 2022;35(1):126–31. 33100090 10.1080/08941939.2020.1836289

[11] Vogel JP, Betrán AP, Vindevoghel N, Souza JP, Torloni MR, Zhang J, et al. Use of the Robson classification to assess caesarean section trends in 21 countries: a secondary analysis of two WHO multicountry surveys. Lancet Glob Health. 2015;3(5):e260–270. 25866355 10.1016/S2214-109X(15)70094-X

[12] Sys D, Kajdy A, Baranowska B, Tataj-Puzyna U, Gotlib J, Bączek G, et al. Women’s views of birth after cesarean section. J Obstet Gynaecol Res. 2021;47(12):4270–9. 34611958 10.1111/jog.15056

[13] Abdelazim I, Alanwar A, Shikanova S, Kanshaiym S, Farghali M, Mohamed M, et al. Complications associated with higher order compared to lower order cesarean sections. J Matern Fetal Neonatal Med. 2020;33(14):2395–402. 30463461 10.1080/14767058.2018.1551352

[14] Biler A, Ekin A, Ozcan A, Inan AH, Vural T, Toz E. Is it safe to have multiple repeat cesarean sections? A high volume tertiary care center experience. Pak J Med Sci. 2017;33(5):1074–9. 29142541 10.12669/pjms.335.12899 PMC5673710

[15] Yaman Tunc S, Agacayak E, Sak S, Basaranoglu S, Goruk NY, Turgut A, et al. Multiple repeat caesarean deliveries: do they increase maternal and neonatal morbidity? J Matern Fetal Neonatal Med. 2017;30(6):739–44. 27125601 10.1080/14767058.2016.1183638

[16] Black M, Bhattacharya S, Philip S, Norman JE, McLernon DJ. Planned Repeat Cesarean Section at Term and Adverse Childhood Health Outcomes: A Record-Linkage Study. Chappell LC, editor. PLOS Med. 2016;13(3):1–16. 10.1371/journal.pmed.1001973 PMC4792387 26978456

[17] Regan J, Thompson A, DeFranco E. The Influence of Mode of Delivery on Breastfeeding Initiation in Women with a Prior Cesarean Delivery: A Population-Based Study. 2013;8(2):181–6. 10.1089/bfm.2012.0049 PMC4209487 23186385

[18] Fawsitt CG, Bourke J, Greene RA, Everard CM, Murphy A, Lutomski JE. At What Price? A Cost-Effectiveness Analysis Comparing Trial of Labour after Previous Caesarean versus Elective Repeat Caesarean Delivery. Derrick GE, editor. PLoS ONE. 2013;8(3):1–8. 10.1371/journal.pone.0058577 PMC3590223 23484038

[19] The American College of Obstetricians and Gynecologists. Vaginal Birth After Cesarean Delivery (VBAC) [Internet]. 2017 [cited 2022 Jan 18]. https://www.acog.org/en/womens-health/faqs/vaginal-birth-after-cesarean-delivery

[20] Habak PJ, Kole M. Vaginal Birth After Cesarean Delivery. In: StatPearls [Internet]. Treasure Island (FL): StatPearls Publishing; 2024 [cited 2024 Sep 26]. http://www.ncbi.nlm.nih.gov/books/NBK507844/29939621

[21] Sabol B, Denman M, Guise J. Vaginal Birth After Cesarean: An Effective Method to Reduce Cesarean. Clin Obstet Gynecol. 2015;58(2):309–19. 25811124 10.1097/GRF.0000000000000101

[22] Lundgren I, van Limbeek E, Vehvilainen-Julkunen K, Nilsson C. Clinicians’ views of factors of importance for improving the rate of VBAC (vaginal birth after caesarean section): a qualitative study from countries with high VBAC rates. BMC Pregnancy Childbirth. 2015;15:1–12. 26314295 10.1186/s12884-015-0629-6 PMC4552403

[23] Lundgren I, Morano S, Nilsson C, Sinclair M, Begley C. Cultural perspectives on vaginal birth after previous caesarean section in countries with high and low rates — A hermeneutic study. Women Birth. 2020;33(4):339–47. 10.1016/j.wombi.2019.07.300 31445846

[24] Nilsson C, van Limbeek E, Vehvilainen-Julkunen K, Lundgren I. Vaginal Birth After Cesarean: Views of Women From Countries With High VBAC Rates. Qual Health Res. 2017;27(3):325–40. 26531882 10.1177/1049732315612041

[25] Lundgren I, Healy P, Carroll M, Begley C, Matterne A, Gross MM, et al. Clinicians’ views of factors of importance for improving the rate of VBAC (vaginal birth after caesarean section): a study from countries with low VBAC rates. BMC Pregnancy Childbirth. 2016;16:1–10. 27832743 10.1186/s12884-016-1144-0 PMC5103375

[26] Nilsson C, Lalor J, Begley C, Carroll M, Gross MM, Grylka-Baeschlin S, et al. Vaginal birth after caesarean: Views of women from countries with low VBAC rates. Women Birth. 2017;30(6):481–90. 28545775 10.1016/j.wombi.2017.04.009

[27] Anekpornwattana S, Yangnoi J, Jareemit N, Boriboonhirunsarn D. Cesarean Section Rate in Siriraj Hospital According to the Robson Classification. Thai J Obstet Gynaecol. 2020;28(1):6–15.

[28] Tamang T, Dema J, Pelden S, Choden P. Usefulness of Robson classification system to analyze caesarean section deliveries: a hospital based study. Bhutan Health J. 2020;6(1):38–44.

[29] Jimenez-Zambrano A, Feller K, Rivera C, Marchin A, Bolanos AG, Asturias E, et al. Perspectives of Obstetricians and Women with a History of Prior Cesarean Birth Regarding Subsequent Mode of Birth in Trifinio and Coatepeque, Guatemala. Obstet Gynecol Res. 2022;5(1):10–9. 35198983 10.26502/ogr074 PMC8863362

[30] Lennon RA, Kearns K, O’Dowd S, Biesty L. VBAC or elective CS? An exploration of decision-making process employed by women on their mode of birth following a previous lower segment caesarean section. Women Birth. 2023;36(6):623–30. 10.1016/j.wombi.2023.05.011 37308355

[31] Lockwood C, Munn Z, Porritt K. Qualitative research synthesis: methodological guidance for systematic reviewers utilizing meta-aggregation. Int J Evid Based Healthc. 2015;13(3):179–87. 26262565 10.1097/XEB.0000000000000062

[32] Chan I, Bronson E, Cantor A. Vaginal Birth after Cesarean Section: Provider Perspectives and Maternal Decision Making. 2015;12:41–8.

[33] Firoozi M, Tara F, Mazlom SR, Latifnejad Roudsari R. A Qualitative Inquiry to Explore Why Women with Previous Cesarean-Section Do Not Choose Vaginal Birth after Cesarean. J Midwifery Reprod Health. 2021;9(2):2753–62.

[34] Foureur M, Turkmani S, Clack DC, Davis DL, Mollart L, Leiser B, et al. Caring for women wanting a vaginal birth after previous caesarean section: A qualitative study of the experiences of midwives and obstetricians. Women Birth. 2017;30(1):3–8. 27318563 10.1016/j.wombi.2016.05.011

[35] Munro S, Kornelsen J, Corbett K, Wilcox E, Bansback N, Janssen P. Do Women Have a Choice? Care Providers’ and Decision Makers’ Perspectives on Barriers to Access of Health Services for Birth after a Previous Cesarean. Birth. 2017;44(2):153–60. 27917532 10.1111/birt.12270

[36] Keedle H, Schmied V, Burns E, Dahlen HG. From coercion to respectful care: women’s interactions with health care providers when planning a VBAC. BMC Pregnancy Childbirth. 2022;22(1):1–14. 35086509 10.1186/s12884-022-04407-6 PMC8793226

[37] Xu X, Lee HC, Lin H, Lundsberg LS, Campbell KH, Lipkind HS, et al. Hospital variation in utilization and success of trial of labor after a prior cesarean. Am J Obstet Gynecol. 2019;220(1):98.e1-98.e14. 10.1016/j.ajog.2018.09.034 30278176

[38] White HK, le May A, Cluett ER. Evaluating a Midwife-Led Model of Antenatal Care for Women with a Previous Cesarean Section: A Retrospective, Comparative Cohort Study. Birth. 2016;43(3):200–8. 26991669 10.1111/birt.12229

[39] Fore MS, Allshouse AA, Carlson NS, Hurt KJ. Outcomes of Trial of Labor After Cesarean Delivery by Provider Type in Low-Risk Women. Birth Berkeley Calif. 2020;47(1):123–34. 31823421 10.1111/birt.12474 PMC7047558

[40] Trinity College Dublin. ScienceDaily. 2018 [cited 2023 Sep 14]. Fear of litigation is a key factor in decision to perform C-sections. https://www.sciencedaily.com/releases/2018/07/180730104859.htm

[41] Deng B, Li Y, Chen JY, Guo J, Tan J, Yang Y, et al. Prediction models of vaginal birth after cesarean delivery: A systematic review. Int J Nurs Stud. 2022;135:1–11. 10.1016/j.ijnurstu.2022.104359 36152466

[42] International Confederation of Midwives. Core Document: Philosophy and Model of Midwifery Care. 2014.

[43] Smith DC, Phillippi JC, Lowe NK, Breman RB, Carlson NS, Neal JL, et al. Using the Robson 10-Group Classification System to Compare Cesarean Birth Utilization Between US Centers With and Without Midwives. J Midwifery Womens Health. 2020;65(1):10–21. 31553129 10.1111/jmwh.13035 PMC7024566

[44] Birara M, Gebrehiwot Y. Factors associated with success of vaginal birth after one caesarean section (VBAC) at three teaching hospitals in Addis Ababa, Ethiopia: a case control study. BMC Pregnancy Childbirth. 2013;13(1):1–6. 23374116 10.1186/1471-2393-13-31 PMC3575257

[45] Wu Y, Kataria Y, Wang Z, Ming WK, Ellervik C. Factors associated with successful vaginal birth after a cesarean section: a systematic review and meta-analysis. BMC Pregnancy Childbirth. 2019;19(1):1–12. 31623587 10.1186/s12884-019-2517-y PMC6798397

[46] Royal College of Obstetrician & Gynaecologists. Birth After Previous Caesarean Birth. 2015.

[47] Toolkit [Internet]. [cited 2023 Sep 9]. Benefits of Guidelines. https://toolkit.ncats.nih.gov/module/after-fda-approval/creating-clinical-care-guidelines/benefits-of-guidelines

[48] Graham R, Mancher M, Wolman DM, Greenfield S, Steinberg E, editors. Clinical Practice Guidelines We Can Trust [Internet]. Washington, D.C.: National Academies Press; 2011 [cited 2023 Nov 17]. https://www.nap.edu/catalog/13058 24983061

[49] Shi M, Zhang H, Huang S, Zhang M, Hu X. Improving the Quality of Maternity Care: Learning From Malpractice. J Patient Saf. 2023;19(4):229–38. 36849439 10.1097/PTS.0000000000001112 PMC10227943

[50] Hadjigeorgiou E, Katsie C, Papadopoulou M, Christofi MD, Christoforou A. Women’s experiences of VBAC in Cyprus: a qualitative study. BMC Pregnancy Childbirth. 2021;21:1–12. 34763658 10.1186/s12884-021-04193-7 PMC8588624

[51] Lazo-Porras M, Bayer AM, Acuña-Villaorduña A, Zeballos-Palacios C, Cardenas-Montero D, Reyes-Diaz M, et al. Perspectives, Decision Making, and Final Mode of Delivery in Pregnant Women With a Previous C-Section in a General Hospital in Peru: Prospective Analysis. MDM Policy Pract. 2017;2(2):1–11. 10.1177/2381468317724409 PMC6125051 30288428

[52] Das M, Varma R. Vaginal birth after caesarean section: a practical evidence-based approach. Obstet Gynaecol Reprod Med. 2012;22(7):177–85.

[53] Maroyi R, Ngeleza N, Keyser L, Bosunga K, Mukwege D. Prenatal care counseling and delivery method among women with multiple Cesareans: A cross-sectional study from Democratic Republic of Congo. PLoS ONE. 2020;15(11):1–9. 10.1371/journal.pone.0238985 PMC7652330 33166279

